# Examining the Impact of Digital Inclusion on Depression Among Older Adults in China: Mediating Role of Noncognitive Abilities

**DOI:** 10.2196/71441

**Published:** 2025-06-27

**Authors:** Xinru Li, Chengyu Chen, Xiyan Li, Yuyang Li, Shujuan Xiao, Jianan Han, Yanan Wang, Chichen Zhang

**Affiliations:** 1 School of Public Health Southern Medical University Guangzhou China; 2 School of Health Management Southern Medical University Guangzhou China; 3 Key Laboratory of Philosophy and Social Sciences of Colleges and Universities in Guangdong Province for Collaborative Innovation of Health Management Policy and Precision Health Service Southern Medical University Guangzhou China; 4 School of Nursing Southern Medical University Guangzhou China

**Keywords:** digital inclusion, depression, Big Five personality, non-cognitive abilities

## Abstract

**Background:**

In the digital and intelligent era, a considerable number of older adults in China still have a low level of digital inclusion. Although existing literature has explored the relationship between the use of the internet and depression among older adults to some extent, it mainly focused on surface aspects rather than delving into the underlying mechanism of action among digital inclusion, depression, and noncognitive abilities, which remains unclear.

**Objective:**

This study aims to explore the mediating role of noncognitive abilities between digital inclusion and depression among older adults in China, as well as the parallel mediating roles of each dimension of the Big Five personality traits in the relationship between them.

**Methods:**

We extracted cross-sectional data from a nationally representative survey, namely the China Family Panel Studies, which centered on older adults (aged 60 years or older). The Center for Epidemiologic Studies Depression Scale (CES-D 8), which consists of 8 items, was used to determine the presence of depression. The 15-item short version of the Big Five Personality Scale was used to measure the noncognitive abilities of older adults. Model 4 of the PROCESS macro (Andrew F. Hayes) program was applied to test, respectively, the mediating role of noncognitive abilities between digital inclusion and depression status, as well as the parallel mediating roles of each dimension of the Big Five personality traits in the relationship between them.

**Results:**

This study found that digital inclusion was negatively related to depression in older adults (*β*=–.054, *t*_6545_=–4.804; *P*<.01). After adding noncognitive abilities as a mediating variable, depression was found to be negatively related to digital inclusion (*β*=–.022, *t*_6544_=–1.972; *P*<.05). Noncognitive abilities play a significant mediating role in the relationship between the level of digital inclusion and depression, and their effect accounts for 59.44% of the total effect. In the parallel mediation model, conscientiousness, extraversion, openness, and emotional stability all partially mediated the association between digital inclusion and depression. The parallel mediation effects of conscientiousness (*β*=–.0045, 95% CI –0.0068 to –0.0024; *P*<.05), extraversion (*β*=–.0067, 95% CI –0.0096 to –0.0043; *P*<.05), openness (*β*=.0085, 95% CI 0.0042 to 0.0128; *P*<.05), and emotional stability (*β*=–.0073, 95% CI –0.0131 to –0.0017; *P*<.05) of noncognitive abilities in the relationship between digital inclusion and depression were significant, accounting for 8.33%, 12.41%, –15.74%, and 13.52% of the total effect, respectively.

**Conclusions:**

Our results demonstrate that digital inclusion is a negative predictor of depression among older adults, and noncognitive abilities play a partial mediating role between digital inclusion and depression status. Moreover, conscientiousness, extraversion, openness, and emotional stability of the Big Five personality traits have parallel mediating effects between digital inclusion and depression status.

## Introduction

### Background

Depression remains one of the most common mental disorders among older adults in China. It severely harms their health and is a leading contributor to late-life mortality. According to the World Health Organization (WHO), over 54.8 million people in China had depression in 2015 [1], accounting for 17% of the global disease burden from mental, neurological, and substance use disorders [2]. The China Aging Development Report (2024): Mental Health Status of Older Adults further highlights that 19.05% of older adults currently experience mild depressive symptoms, while 12.17% exhibit moderate to severe symptoms [3]. Notably, the report identifies digital lifestyles as a positive factor in reducing loneliness with frequent internet users reporting the lowest loneliness rates [3]. These findings suggest that internet engagement may offer a new pathway to improve mental health in aging populations.

The rapid growth of the digital economy is transforming human behavior patterns [4]. For older adults, the internet helps address lifestyle limitations caused by declining physical functions. Features such as health information access, remote social interactions, and digital entertainment support healthy aging. Studies confirm that internet use significantly improves health outcomes in older adults [5], but scholarly debates persist. Some scholars warn that excessive reliance on internet-based information may increase mental health risks [6]. Older adults also face challenges in adapting to digital transitions, such as skill gaps in mobile payments and internet-based fraud detection [7], as well as intergenerational cultural divides linked to differences in new media use [8]. Hong et al [9] revealed a paradox despite rising internet penetration in China, digital access remains concentrated among high socioeconomic groups and shows complex ties to older adults’ health. This “digital divide” has become a key social determinant of mental health, particularly depression, in older populations [10].

To tackle the digital divide, scholars propose “digital inclusion”—a concept focused on narrowing digital gaps by improving device accessibility and enhancing digital skills among vulnerable groups [11]. Existing research often examines how digital exclusion affects cognitive abilities. For example, Wang et al [12] found that 97.15% of Chinese older adults experience digital exclusion, which strongly correlates with cognitive impairment. However, cognitive abilities show relative stability in early life [13], although they can adapt to individual experiences in middle and later life [14]. In contrast, noncognitive abilities exhibit greater plasticity over time [15]. This has shifted research attention to noncognitive abilities, such as personality traits. Studies show that these abilities remain adaptable in later life, compensating for health disadvantages in vulnerable groups [16], and improving health outcomes by promoting healthy behaviors and social engagement [17]. These insights provide new theoretical perspectives for understanding how digital inclusion influences mental health.

Despite progress, critical gaps remain in understanding how noncognitive abilities mediate the relationship between digital inclusion and late-life depression. First, existing studies show inconsistent conclusions, requiring more systematic frameworks. Second, most research oversimplifies digital inclusion by using binary “internet use” indicators. Third, few studies explore the multidimensional mechanisms of noncognitive abilities. To address these gaps, this study develops a 3D digital inclusion framework (access, usage, and literacy) and integrates the Big Five personality theory. By uncovering new protective mechanisms for mental health in the digital age, this research aims to guide targeted interventions for older adults.

### Conceptual Definition and Hypotheses

#### Digital Inclusion

The rapid advancement of digital technology has generated substantial societal benefits. However, its widespread adoption has increasingly marginalized vulnerable groups, particularly older adults, who face difficulties accessing inclusive digital resources and experience challenges in adapting to digital life. The growing visibility of the “digital divide” has drawn public concern, prompting efforts to reduce disparities between groups. Digital inclusion mechanisms have emerged as critical strategies to mitigate digital exclusion. Broadly defined, “digital inclusion” refers to actions and processes that bridge the digital divide [11]. From a microlevel perspective, it encompasses dimensions such as household access to digital devices and the technological proficiency of disadvantaged groups [7]. This concept transcends traditional binary measurements (eg, internet use vs nonuse), emphasizing multilayered adaptation, from digital access and usage skills to digital literacy, providing a framework to evaluate older adults’ adaptability in digital societies.

#### Noncognitive Abilities and the Big Five Personality Traits

Noncognitive abilities, as key psychological characteristics, describe stable and enduring patterns of thinking, emotional responses, and behaviors in specific contexts [18]. Personality psychology research confirms that these abilities align with the Big Five personality model, which categorizes traits into five dimensions: neuroticism, agreeableness, conscientiousness, openness, and extraversion [19]. Unlike cognitive abilities with standardized assessments, noncognitive abilities lack consensus in measurement due to their diverse and context-dependent nature [20].

Common measurement tools include the Locus of Control Scale, Self-Esteem Scale, and the Big Five Inventory (BFI). The BFI, favored for its comprehensive structure and cross-cultural validity, serves as the primary tool in this study. We use the abbreviated Neuroticism, Extraversion, Openness Five-Factor Inventory (NEO-FFI) to quantify noncognitive abilities in older adults, where higher scores across dimensions reflect stronger ability development.

Critically, studies highlight distinct effects of personality dimensions on depressive symptoms. Clinical evidence links high neuroticism, low conscientiousness, and emotional instability to increased depression risk [21]. This dimension-specific effect suggests personality traits may mediate the relationship between digital inclusion and mental health. To explore this, we design a parallel mediation model to dissect how each Big Five dimension influences the pathway between digital technology use and depressive states, thereby revealing psychological adaptation mechanisms.

### Digital Inclusion and Depression

Recent studies demonstrate that internet use alleviates depressive symptoms in older adults. Heo et al [22] found that frequent internet use predicts stronger social support, reduced loneliness, and better mental health through structural equation modeling. Shapira et al [23] confirmed via social experiments that older adults using computers and the internet exhibit lower depression and loneliness, alongside higher life satisfaction. Wang et al [24] used propensity score matching to show that Chinese older adults with internet access report significantly lower depression levels than nonusers, with prolonged use further reducing symptoms [25].

Although previous studies have achieved significant progress, current research still exhibits notable limitations in measurement approaches. Most existing studies simplify internet usage as a binary variable (internet-based or offline status). This approach fails to comprehensively capture the complex characteristics of digital engagement among older adults. In contrast, digital inclusion serves as a comprehensive indicator of internet engagement. It encompasses multiple dimensions including access to devices, operational skills, and digital literacy [26], providing a more accurate reflection of older adults’ digital adaptation. However, few studies have systematically investigated the mechanisms linking digital inclusion to geriatric depression. Building on this foundation, this study adopts a digital inclusion theoretical framework. Following established methodologies [27], we develop a multidimensional measurement model incorporating digital access, usage proficiency, and literacy competencies. Based on this analytical framework, we propose the following: Hypothesis 1—digital inclusion levels negatively predict depressive symptoms in older adults; specifically, higher digital inclusion corresponds to lower scores on depression scales.

### The Mediating Role of Noncognitive Capacities Between Digital Inclusion and Depression

Noncognitive capacities constitute a critical dimension of human capital, encompassing emotional regulation, psychological resilience, and behavioral patterns [28]. Focusing on digital technology pathways, Li and Sun [29] demonstrated that internet use enhances noncognitive capacities. However, their findings revealed selective effects, with digital engagement significantly improving extraversion and openness while showing negligible impacts on conscientiousness, agreeableness, or emotional stability. This heterogeneous effect likely stems from digital technology’s distinct mechanisms. The internet breaks spatial-temporal barriers to information access and provides cross-cultural interaction platforms, exposing users to diverse values. These processes collectively stimulate cognitive flexibility and reshape thinking patterns. Digital inclusion holds unique value for older adults’ noncognitive development. It accelerates intergenerational cultural transmission and expands social participation opportunities. These dual pathways address two critical challenges: mitigating social isolation and reconstructing emotional identity. Together, they establish self-reinforcing cycles that promote mental well-being.

Although existing literature has not directly explored noncognitive capacities’ mediating role in depression in older adults, Big Five personality research offers critical insights. Cai et al [30] identified significant negative correlations between depression and four traits in Chinese adults: extraversion, agreeableness, conscientiousness, and emotional stability. Koorevaar et al [31] observed similar patterns in Dutch older adults. Their study highlighted 3 key associations. First, depression severity correlates with higher neuroticism and lower extraversion or conscientiousness. Second, early-onset depression is linked to elevated openness. Third, no significant relationship exists between depression and agreeableness [31].

Based on this theoretical and empirical foundation, we propose the following:

Hypothesis 2: noncognitive capacities mediate the relationship between digital inclusion and depression in older adults.

Hypothesis 3: the mediating effects exhibit significant heterogeneity across personality dimensions.

## Methods

### Study Design and Participants

**Figure 1:**
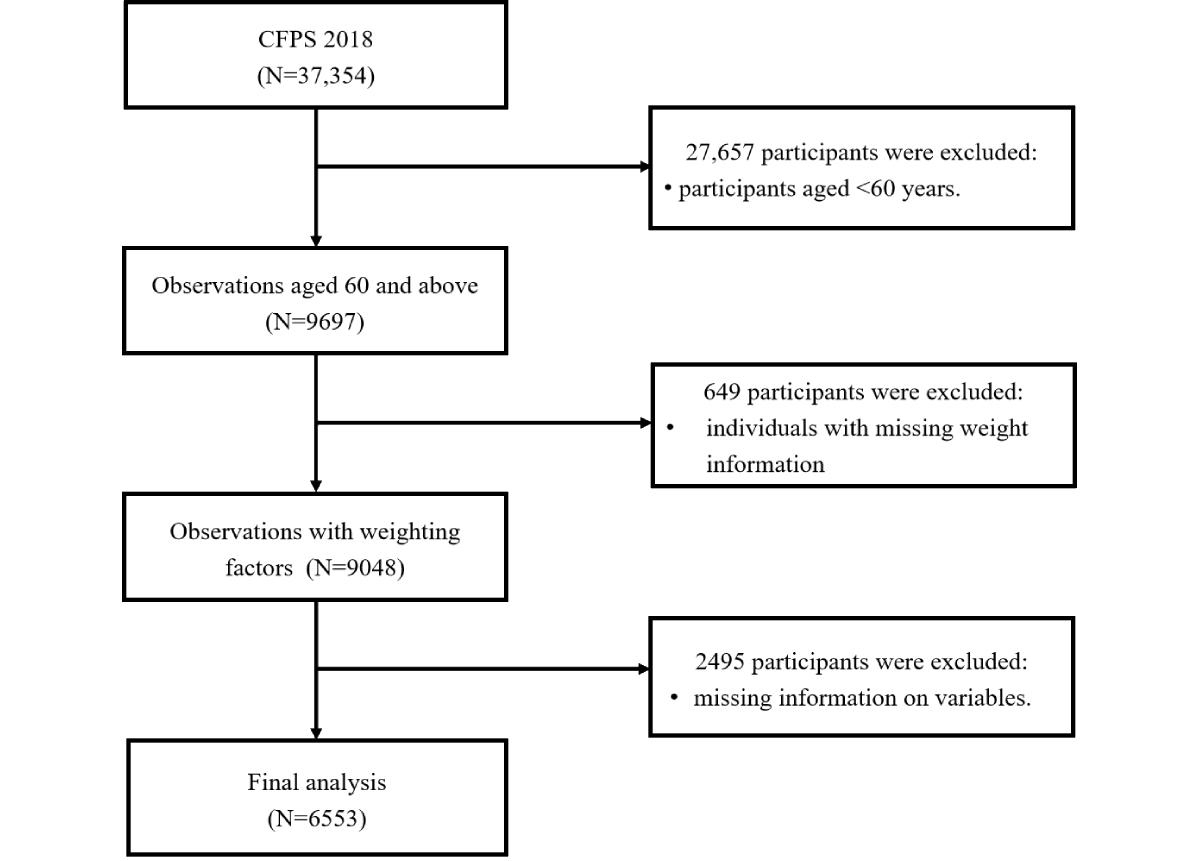
Selection of participants. CFPS: China Family Panel Studies.

The data used in this study were derived from the China Family Panel Studies (CFPS), a nationally representative and comprehensive longitudinal survey conducted by Peking University. The CFPS covers a broad spectrum of research topics, including economic activities, educational attainment, family relationships and dynamics, population migration, and physical and mental health. The baseline survey was initiated in 2010 and included a sample of 42,590 individuals from 25 provinces, municipalities, and autonomous regions across China. These individuals form the permanent tracking cohort of the CFPS and are interviewed biennially. Since the comprehensive measurement of noncognitive abilities was first introduced in the 2018 wave of the CFPS, this study uses the 2018 data. The sample selection process is depicted in Figure 1.

In 2018, the CFPS collected data from 37,354 individuals. This study included participants aged 60 years and older but excluded those who were cognitively impaired or unwilling to participate. We removed cases with missing weight values from the sample and applied Little’s Missing Completely at Random multivariate test to assess the pattern of missing data. The results showed a missing data pattern (Χ^2^_1_=0.001; *P*=.97), confirming that the missing data were completely random. Existing research suggests that when data are missing completely at random, excluding cases with missing data does not introduce bias into the overall dataset [32]. Therefore, after removing individuals with missing values in key variables, we retained a final sample of 6553 individuals for analysis.

### Measures

#### Digital Inclusion

Current research lacks a standardized definition for measuring digital inclusion. Operational variables vary across studies due to divergent data sources. However, the 3-tier framework, aligning with the digital divide’s access, usage, and literacy gaps, remains the most widely adopted approach [7,27]. For instance, Jiang and Shi [33] conceptualized digital inclusion using these 3 dimensions when investigating its mediating role between aging attitudes and mental health. This study adopts the same framework.

#### Access-Level Measurement

This tier evaluates older adults’ physical access to the internet. Participants receive 0 points if they answer “no” to both smartphone and computer internet use. Otherwise, they receive 1 point.

#### Usage-Level Measurement

This tier examines structural disparities in internet skills and frequency. Following established protocols [27], we categorize digital skills into two types: basic recreational skills (eg, entertainment) and advanced value-creation skills (eg, learning and productivity). Participants report their frequency of internet use for 5 activities: learning, work, socializing, entertainment, and commercial activities. Responses range from “never” (0) to “almost daily” (6).

#### Literacy-Level Measurement

This tier assesses critical understanding and cultural awareness in digital engagement. Adopting previous methodologies [34], we measure this dimension using 4 items from the CFPS survey: (1) the importance of television for information acquisition, (2) the importance of the internet for information acquisition, (3) the importance of SMS text messaging for information acquisition, and (4) importance of radio for information acquisition. Responses are assessed on a 5-point Likert scale ranging from 0 (“not important at all”) to 4 (“extremely important”).

The dependent variable is calculated by summing scores from the 3 tiers (access, usage, and literacy). Total scores range from 0 to 47 with higher scores indicating better digital inclusion among older adults.

#### Depression

We used the shortened 8-item version of the Center for Epidemiologic Studies Depression Scale (CES-D). Multiple studies have validated its applicability for assessing mental health in older Chinese populations [35]. The 2018 CFPS questionnaire includes this scale with items 4 and 6 reverse-scored. Scores range from 0 to 24, where higher values indicate more severe depressive symptoms. We evaluated the scale’s reliability and validity. Results demonstrated strong internal consistency (Cronbach α=0.79). The Kaiser-Meyer-Olkin measure reached 0.816, and the Bartlett sphericity test yielded a chi-square value of 13846.7 (*df*=28; *P*<.001), confirming robust construct validity.

#### Noncognitive Abilities

This study uses the Big Five Personality Inventory to measure noncognitive abilities. Widely recognized for its comprehensive personality assessment, this instrument has been validated across World Bank surveys and studies in Germany, Poland, and the United Kingdom [36,37]. It also shows strong reliability in older adult populations [38,39]. The 2018 CFPS dataset incorporates a 15-item abbreviated version assessing 5D: conscientiousness, extraversion, agreeableness, openness, and neuroticism. Items use a 5-point Likert scale. To ensure consistent directional effects, we recoded neuroticism as emotional stability. Following methodological precedents [40,41], we omitted Cronbach α calculations due to the brevity of subscales (3 items per dimension).

#### Covariate

Drawing on previous research [42], we controlled for age, self-rated health, pension status, filial support, marital status, and chronic disease presence. Table 1 details variable coding procedures.

**Table 1:** Sample characteristics (mean or percentage).

Variable		Full sample (N=6553)	Female (n=3228)	Male (n=3325)
**Continuous variables, mean (SD)**				
	Digital inclusion	5.56 (4.72)	4.73 (3.95)	6.36 (5.24)
	Depression	5.95 (4.55)	6.74 (4.73)	5.19 (4.25)
	Noncognitive abilities	52.13 (6.18)	51.54 (6.04)	52.71 (6.26)
Age (years), mean (SD)		68.21 (6.32)	68.17 (6.28)	68.24 (6.36)
**Self-rated health,** **n (%)**				
	1=Very healthy	561 (8.56)	257 (7.96)	304 (9.14)
	2=Relatively healthy	612 (9.34)	265 (8.21)	347 (10.44)
	3=Average	2300 (35.10)	991 (30.70)	1309 (39.37)
	4=Relatively poor	1116 (17.03)	541 (16.76)	575 (17.29)
	5=Very poor	1964 (29.97)	1174 (36.37)	790 (23.76)
**Marital status,** **n (%)**				
	0=nonmarried (unmarried, cohabiting, and divorced)	153 (2.34)	46 (1.43)	107 (3.22)
	1=married (with a spouse)	5306 (80.97)	2405 (74.50)	2901 (87.25)
	2=widowed	1094 (16.69)	777 (24.07)	317 (9.53)
**Chronic disease,** **n (%)**				
	0=no	4580 (69.89)	2141 (66.33)	2439 (73.35)
	1=yes	1973 (30.11)	1087 (33.67)	886 (26.65)
**Pension, n (%)**				
	0=no	1906 (29.09)	988 (30.61)	918 (27.61)
	1=yes	4647 (70.91)	2240 (69.39)	2407 (72.39)
**Household registration status,** **n (%)**				
	0=agricultural household registration	4929 (75.22)	2459 (76.18)	2470 (74.29)
	1=nonagricultural household registration	1616 (24.66)	765 (23.70)	851 (25.59)
	2=others	8 (0.12)	4 (0.12)	4 (0.12)

### Data Analysis

The data analysis was carried out by means of SPSS (version 27.0; IBM Corp) and the PROCESS macro (version 4.2, Andrew F. Hayes). Initially, the Mann-Whitney *U* test or the Kruskal-Wallis H test was used to scrutinize the disparities in digital inclusion and depressive states with respect to diverse demographic features. Subsequently, the Spearman correlation analysis was used to probe into the correlations among the principal variables. Third, Model 4 of the PROCESS macro program was deployed to separately examine the mediating effect of noncognitive abilities between digital inclusion and depressive states, as well as the parallel mediating effects of each dimension of the Big Five personality traits within this relationship. Data weighting procedures were administered preceding mediation analysis to address sampling design characteristics. Eventually, the bootstrapping approach with 5000 resamples and a 95% CI was used to assess the statistical significance of the indirect effects.

### Ethical Considerations

Ethical approval for all waves of CFPS was granted by the institutional review board at Peking University. The institutional review board approval number for the primary household survey, inclusive of anthropometric measurements, is IRB00001052-14010. Informed consent was obtained in written form from all participants at the time of their enrollment. All data collected in the CFPS has been anonymized and deidentified to protect the privacy of participants. There is no personally identifiable information in the released dataset. No additional ethical clearance was required for users of the approved data.

## Results

### Descriptive Statistics

The study included 6553 participants (3325 males and 3228 females) with a mean age of 68.21 (SD 6.32) years. Older adults demonstrated an average digital inclusion score of 5.56 (SD 4.72) and an average depressive symptom score of 5.95 (SD 4.55). Detailed sample characteristics are presented in Table 1. Univariate analyses of digital inclusion and depressive symptoms are summarized in Table 2. For digital inclusion, significant differences emerged across age (*H*=69.04; *P*<.01), sex (*Z*=–15.17; *P*<.01), self-rated health (*H*=105.22; *P*<.01), marital status (*H*=82.30; *P*<.01), pension status (*Z*=–3.67; *P*<.01), and household registration (*H*=109.45; *P*<.01). No significant difference was observed for chronic disease status (*Z*=–0.49; *P*>.05). Depressive symptoms showed significant variations by sex (*Z*=–13.98; *P*<.01), self-rated health (*H*=869.08; *P*<.01), marital status (*H*=172.05; *P*<.01), chronic disease presence (*Z*=–13.21; *P*<.01), pension status (*Z*=–3.91; *P<*.01), and household registration (*H*=147.22; *P*<.01). Age exhibited no significant association (*H*=1.99; *P*>.05).

**Table 2:** Differences in the digital inclusion level and depressive state of older adults under different demographic characteristics.

Variable		Digital inclusion				Depression		
		*Z* ^a^		*H* ^b^		*Z*		*H*
**Age (years)**		—^c^		69.04^d^		—		1.99
	60-65							
	66-70							
	71-75							
	76-80							
	81 or older							
**Sex**		–15.17^d^	—		–13.98^d^		—	
	Female							
	Male							
**Self-rated health**		—	105.22^d^		—		869.08^d^	
	Very healthy							
	Relatively healthy							
	Average							
	Relatively poor							
	Very poor							
**Marital status**		—	82.30^d^		—		172.05^d^	
	Married (with a spouse)							
	Nonmarried (unmarried, cohabiting, and divorced)							
	Widowed							
**Chronic disease**		–0.49	—		–13.21^d^		—	
	No							
	Yes							
**Pension**		–3.67^d^	—		–3.91^d^		—	
	No							
	Yes							
**Household registration status**		—	109.45^d^		—		147.22^d^	
	Agricultural household registration							
	Nonagricultural household registration							
	Others							

^a^Z represents the Mann-Whitney *U* test.

^b^H represents the Kruskal-Wallis H test.

^c^Not applicable.

^d^*P*<.01.

### Common Method Bias Test

We assessed common method bias using the Harman single-factor test. The analysis identified 8 factors with eigenvalues exceeding 1. The first factor accounted for 12.936% of the variance, below the critical threshold of 40%. These results indicate no substantial common method bias in this study.

### Correlation Analysis of Main Variables

Following normality tests, we found that all key variables violated normal distribution assumptions. We therefore conducted Spearman correlation analyses to examine relationships among digital inclusion levels, depressive symptoms, and noncognitive abilities. Table 3 displays these results. A negative correlational relationship was observed between the digital inclusion level of the older population and their depressive state (*r*=–0.102, *P*<.01), signifying that as the digital inclusion level ascended, the depressive state of older adults exhibited a downward tendency. Likewise, a positive correlational association was detected between digital inclusion and noncognitive abilities (*r*=0.199, *P*<.01), implying that with the augmentation of noncognitive abilities among older adults, the digital inclusion level also demonstrated an upward inclination. Furthermore, a negative correlation was identified between the noncognitive abilities of older adults and depression (*r*=–0.305, *P*<.01). That is to say, when the noncognitive abilities were enhanced, the depressive state of older adults manifested a decreasing trend.

**Table 3:** Correlations between the main variables.

Variable	Mean (SD)	Digital inclusion (*r*)	Depression (*r*)	Noncognitive abilities (*r*)
Digital inclusion	5.56 (4.72)	1.000	–0.102^a^	0.199^a^
Depression	5.95 (4.55)	–0.102^a^	1.000	–0.305^a^
Noncognitive abilities	52.13 (6.18)	0.199^a^	–0.305^a^	1.000

^a^*P*<.01.

### Examination of the Mediating Effects of Noncognitive Abilities Between Digital Inclusion and Depression

Model 4 of the PROCESS module within the SPSS macro was used to conduct the mediating effect test. Upon controlling for variables including age, self-rated health status, pension availability, filial support, marital status, and the presence of chronic diseases, a mediation model was established to examine the underlying relationships. In this model, the digital inclusion level of older adults served as the predictor variable, the noncognitive abilities were designated as the mediating variable, and the depression level was regarded as the outcome variable. The bootstrap method was implemented with 5000 repeated samplings performed with replacement. The significance was evaluated by determining whether the 95% CI encompassed the value of 0. The results are presented in Table 4. The findings revealed that there existed a negative correlation between digital inclusion and depression among older adults (*β*=–.054, *t*_6545_=–4.804; *P*<.01). After introducing noncognitive abilities as a mediating variable, it was observed that depression was inversely related to digital inclusion (*β*=–.022, *t*_6544_=–1.972; *P*<.05), and non-cognitive abilities exhibited a positive association with digital inclusion (*β*=.204, *t*_6545_=12.864; *P*<.01). Additionally, noncognitive abilities emerged as a significant negative predictor of depression (*β*=–.157, *t*_6544_=–18.427; *P*<.01). In the bias-corrected percentile bootstrap analysis, the mediating effect of noncognitive abilities on the relationship between the levels of digital inclusion and depression was found to be statistically significant, accounting for 59.44% of the total effect. Consequently, it can be concluded that noncognitive abilities partially mediated the relationship between digital inclusion and depression.

**Table 4:** Testing noncognitive abilities as a mediator in the relationship between digital inclusion and depression.

Criterion	Predictors	*R* ^2^	*β*	SE	*t* test (*df*; 95% CI)
Depression	Digital inclusion	0.144	–.054	0.011	–4.804^a^ (6545; –0.076 to –0.032)
Noncognitive abilities	Digital inclusion	0.072	.204	0.016	12.864^a^ (6545; 0.173 to 0.235)
Depression	Digital inclusion	0.186	–.022	0.011	–1.972^b^ (6544; –0.044 to –0.001)
Depression	Noncognitive abilities	0.186	–.157	0.009	–18.427^a^ (6544; –0.174 to –0.141)

^a^*P*<.01.

^b^*P*<.05.

### Test of Parallel Mediation Model

**Figure 2:**
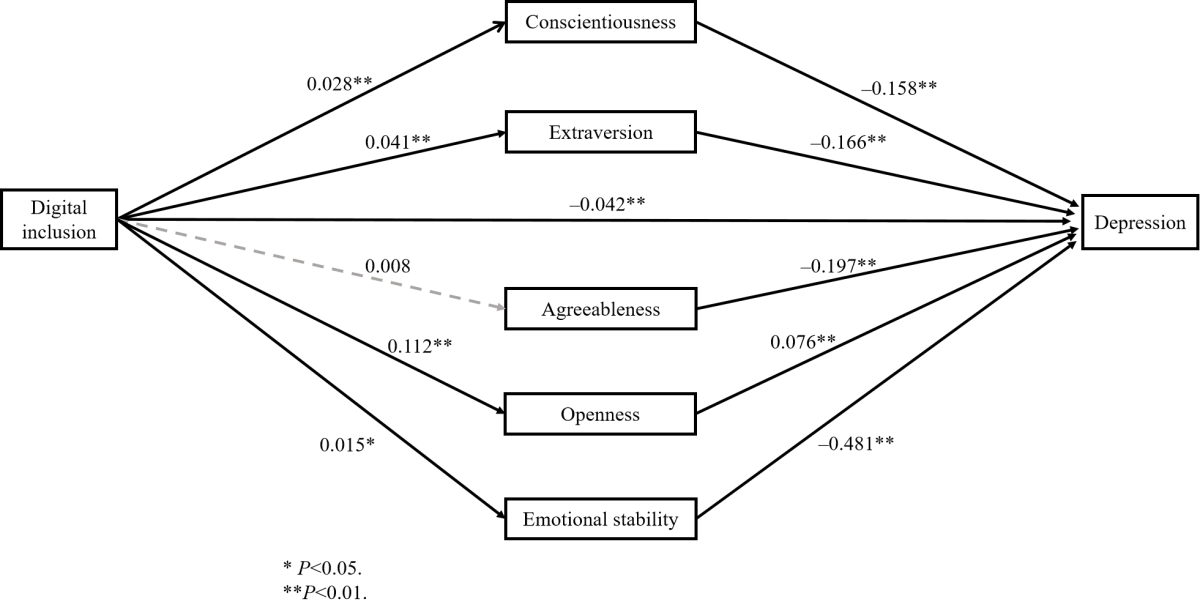
The parallel mediation model for each dimension of noncognitive abilities.

A parallel mediating model was used to explore the potential mediating effects within the relationship between digital inclusion and depression. Consequently, the bootstrap test was implemented to validate the parallel mediating functions of each dimension of the Big Five personality traits (as illustrated in Figure 2). The findings (Table 5) revealed that the parallel mediating effects of conscientiousness, extraversion, openness, and emotional stability of noncognitive abilities in the connection between digital inclusion and depression were statistically significant. Within the parallel mediating model, the overall effect of digital inclusion on geriatric depression was pronounced (*β*=–.0540, 95% CI –0.0761 to –0.0320; *P*<.01). The direct effect of digital inclusion on geriatric depression was also notable (*β*=–.0423, 95% CI –0.0635 to –0.0211; *P*<.01), constituting 78.83% of the total effect. The indirect effect of digital inclusion on depression via the conscientiousness aspect of noncognitive abilities was significant (*β*=–.0045, 95% CI –0.0068 to –0.0024; *P*<.05), accounting for 8.33% of the total effect. The indirect effect of digital inclusion on depression through the extraversion dimension of noncognitive abilities was significant as well (*β*=–.0067, 95% CI –0.0096 to –0.0043; *P*<.05), representing 12.41% of the total effect. The indirect effect of digital inclusion on depression by means of the emotional stability of noncognitive abilities was significant (*β*=–.0073, 95% CI –0.0131 to –0.0017; *P*<.05), making up 13.52% of the total effect. In summary, conscientiousness, extraversion, and emotional stability all partially mediated the correlation between digital inclusion and depression. Additionally, openness exerted a suppressive effect on the pathway from digital inclusion to depression (*β*=.0085, 95% CI 0.0042 to 0.0128; *P*<.05), accounting for –15.74% of the total effect.

**Table 5:** The findings from parallel mediation model tests.

Model pathways		*β*	SE	95% CI
Direct effect Di^a^→Dn^b^		–.0423	0.0108	–0.0635 to –0.0211
**Indirect effect 1**				
	Di→Cs^c^→Dn	–.0045	0.0011	–0.0068 to –0.0024
**Indirect effect 2**				
	Di→En^d^→Dn	–.0067	0.0014	–0.0096 to –0.0043
**Indirect effect 3**				
	Di→As^e^→Dn	–.0016	0.0010	–0.0036 to 0.0002
**Indirect effect 4**				
	Di→Os^f^→Dn	.0085	0.0022	0.0042 to 0.0128
**Indirect effect 5**				
	Di→Nm^g^→Dn	–.0073	0.0029	–0.0131 to –0.0017
Total effect Di→Dn		–.0540	0.0112	–0.0761 to –0.0320

^a^Di: Digital inclusion.

^b^Dn: Depression.

^c^Cs: Conscientiousness.

^d^En: Extraversion.

^e^As: Agreeableness.

^f^Os: Openness.

^g^Nm: Neuroticism.

## Discussion

### Principal Findings

Drawing on 2018 CFPS data, we systematically elucidated the mechanisms linking digital inclusion to reduced depressive symptoms among older adults through simple and parallel mediation models. Our analysis demonstrates that digital inclusion directly alleviates depression while also operating through dual pathways: noncognitive abilities and multidimensional Big Five personality traits. These findings advance theoretical frameworks for mental health interventions in aging populations within digital societies and provide actionable insights for policy makers.

By transcending traditional “technology access” paradigms, we constructed a comprehensive digital inclusion index spanning access, usage, and literacy. Our results reinforce the conclusions of Liu et al [10] about internet skill development mitigating depression. Pandemic case studies revealed that limited digital literacy prevented older adults from accessing telemedicine and digital social interactions, significantly worsening anxiety and depressive symptoms [43]. Crucially, high digital inclusion reflects not only technical competence but also proactive knowledge acquisition and social engagement—dual psychological attributes that likely drive its depression-reducing effects.

Further analysis identified noncognitive abilities as critical mediators. Integrating Yang and Chu’s [28] digital literacy framework with the media intervention model of Chen et al [38], we pioneer evidence of their mediating role in holistic digital inclusion. Digital engagement enhances psychological resilience through three pathways: remodeling social interactions, optimizing learning strategies, and diversifying recreational activities. Smart devices’ instant feedback mechanisms appear particularly impactful, potentially boosting self-efficacy through accumulated success experiences—a process aligning with Bandura’s social cognitive theory. We propose embedding psychological capital development into digital training programs by implementing tiered learning objectives to foster achievement motivation and designing social tasks to improve emotional expression.

The Big Five personality traits revealed heterogeneous mediating effects. Openness showed the strongest negative mediation (15.74%), contrasting with the association between high openness and early-onset depression reported by Koorevaar et al [31]. This paradox may stem from digital environments simultaneously enabling knowledge exploration and inducing cognitive overload through information overexposure. Tailored interventions are warranted. These may include information-filtering training for highly open individuals, stress-reduction interfaces for neuroticism-prone users, and social modeling initiatives leveraging extraverts’ influence. Although conscientiousness exhibited the weakest mediation (8.33%), its potential long-term regulatory effects through improved digital self-management merit further investigation.

The findings highlight the importance of incorporating targeted interventions into community programs to enhance older adults’ conscientiousness, extraversion, and emotional stability. Specifically, we recommend implementing psychological adjustment programs that include breathing exercises and relaxation training. These interventions can strengthen emotional stability by reducing anxiety and improving stress management capabilities. Organizing online interest groups focused on activities such as digital painting may further support emotional regulation through enjoyable skill-building experiences. Furthermore, structured guidance on using social platforms such as WeChat (Tencent) for maintaining connections with family and friends could effectively foster extraversion by increasing social engagement. Additionally, encouraging older adults to participate in community volunteer initiatives, such as mentoring peers with limited digital skills, may enhance conscientiousness through purposeful role fulfillment and skill-sharing opportunities. Future studies should further investigate the dose-response relationship between digital inclusion efforts and personality development while establishing optimized intervention models tailored to specific personality trait profiles.

### Contributions and Implications

Building upon previous research, this study delved further into the relationships among digital inclusion, depression, and noncognitive abilities in older adults. Additionally, it investigated the parallel mediating effects of each dimension of the Big Five personality traits between digital inclusion and depression. The findings of this study reveal that (1) digital inclusion serves as a negative predictor of depression among older adults; that is to say, a greater level of engagement by older adults in the digital realm is potentially conducive to reducing their susceptibility to depression; (2) in the process of enhancing the digital inclusion of older adults to prevent or alleviate depressive moods, particular attention should be directed toward fortifying their noncognitive capabilities; and (3) when strengthening the noncognitive abilities of older adults, priority should be assigned to the 3 personality traits of conscientiousness, extraversion, and emotional stability within the scope of noncognitive abilities.

The findings of this research hold substantial significance in ameliorating the issue of depression among older adults. We have corroborated the negative influence of digital inclusion on geriatric depression and unearthed the mediating function of noncognitive abilities therein. These findings are capable of furnishing novel perspectives and approaches for future mental health interventions. Moreover, it has been ascertained that not all dimensions of the Big Five personality traits serve as mediators between digital inclusion and depression. This discovery delineates the key areas for the subsequent implementation of digital inclusion initiatives, averting the squandering of resources that might otherwise occur due to haphazard execution and the consequent failure to attain the anticipated outcomes.

The results of this study have important implications for policy makers, health care providers, and community organizations. First, efforts to promote digital inclusion among older adults should be prioritized, including providing accessible digital literacy training, developing age-friendly technologies, and fostering supportive social networks. Second, interventions aimed at enhancing noncognitive abilities, such as emotional regulation and social skills, could serve as effective strategies to alleviate depressive symptoms. Furthermore, personalized approaches that consider individual personality traits may improve the efficacy of mental health interventions.

### Limitations

Our study has several noteworthy limitations. First, the cross-sectional design restricts causal inference and obscures dynamic interactions between digital engagement and depression and depressive symptoms may reciprocally reduce digital engagement through motivation depletion. This potential bidirectionality necessitates longitudinal verification using cross-lagged panel models or ecological momentary assessments. Second, while validated, the abbreviated Big Five inventory risks cultural bias by potentially overlooking collectivist-oriented traits critical to Chinese older adults’ digital socialization, such as familial responsibility. Future studies should integrate longitudinal or experimental designs and develop more culturally grounded personality instruments to clarify digital inclusion’s causal mechanisms on mental health and examine noncognitive abilities’ mediating roles.

### Conclusion

Using CFPS data, this study shows that older adults with greater digital inclusion have lower depression risks with enhanced noncognitive abilities strengthening this protective effect. Increased conscientiousness, extraversion, and emotional stability boost digital inclusion’s depression-predictive power, while openness unexpectedly reduces it. These results demonstrate two mental health pathways, such as general skill growth and personality-specific adaptation, for digital engagement’s protective effects. As digitalization accelerates, older adults face worsening mental health challenges from technological exclusion, including anxiety and depression due to digital divides. These urgent issues require societal action. Future work should clarify why openness weakens digital inclusion’s benefits, test culturally adapted interventions, and develop technologies that align digital access with personality differences to support equitable mental health in aging populations.

## Abbreviations

BFI: Big Five Inventory
CES-D: Center for Epidemiologic Studies Depression Scale
CFPS: China Family Panel Studies
NEO-FFI: NEO Five-Factor Inventory
WHO: World Health Organization

## Data Availability

The datasets generated or analyzed during this study are not publicly available due to other related undergoing study but are available from the corresponding author on reasonable request.
